# Determination of halogenated hydrocarbons in urine samples using a needle trap device packed with Ni/Zn–BTC bi-MMOF *via* the dynamic headspace method

**DOI:** 10.1039/d1ra03227e

**Published:** 2021-06-17

**Authors:** Razzagh Rahimpoor, Ali Firoozichahak, Davood Nematollahi, Saber Alizadeh, Parsa Mohammad Alizadeh, Ali Akbar Alinaghi Langari

**Affiliations:** Department of Occupational Health Engineering, Research Center for Health Sciences, School of Health, Larestan University of Medical Sciences Larestan Iran; Department of Occupational Health, Faculty of Health, Social Determinants of Health Research Center, Gonabad University of Medical Science Gonabad Iran a.firoozi@edu.umsha.ac.ir; Department of Chemistry, Bu-Ali-Sina University Hamedan Iran s.alizadeh93@basu.ac.ir s.alizade66@yahoo.com; Department of Occupational Health Engineering, Faculty of Medical Sciences, Tarbiat Modares University Tehran Iran; Student Research Committee, School of Public Health, Bam University of Medical Sciences Bam Iran aliakbarlangari@yahoo.com

## Abstract

In this study, a nickel/zinc–BTC bi-metallic metal–organic framework (bi-MMOF) was employed as a new and efficient adsorbent in a needle trap device (NTD) for headspace (HS) sampling, extraction and analysis of halogenated hydrocarbons (trichloroethylene, tetrachloroethylene, chloroform, and tetrachloroethylene) from spiked and real urine samples. Characterization of the prepared adsorbent was accomplished by FT-IR, PXRD, EDX, elemental mapping, and FE-SEM techniques. According to experimental results, the optimal temperature and extraction time, salt content, temperature and desorption time of the response surface methodology (RSM) and Box–Behnken design (BBD) were determined to be 56 °C and 30 min, 5.5%, 350 °C and 8 min for the studied halogenated hydrocarbons, respectively. The calculated values of detection limit and quantitation limit parameters were in the range of 1.02–1.10 and 2.01–2.4.0 ng L^−1^, respectively. Moreover, intermediate precision and repeatability of the method were in the range of 4.90–8.20% and 1.50–4.80%, respectively. The recovery percentages of analytes were obtained to be in the range of 95.0–97.0% 10 days after the sampling and storage at 4 °C. This study showed that the proposed HS-NTD:Ni/Zn–BTC method coupled with a GC-FID can be employed as a simple, fast, and sensitive procedure for non-metabolized halogenated hydrocarbons from urine samples in biological monitoring.

## Abstract 2

Designed setup for sampling of halogenated hydrogens in urine using the HS-NTD:Ni/Zn–BTC bi-MMOF.

## Graphical abstract

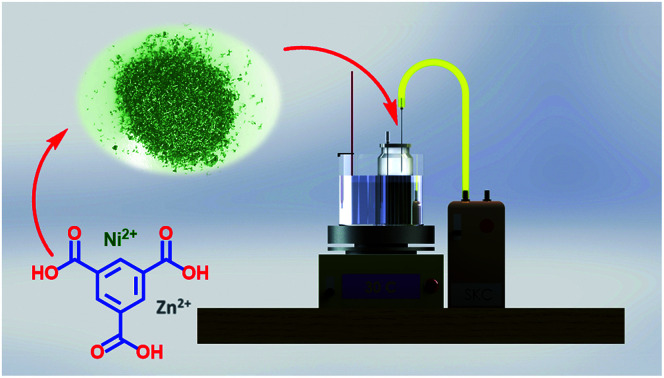

## 1. Introduction

Halogenated hydrocarbons are an important group of volatile compounds that are widely used in industrial applications as solvents, degreasing agents, detergents, and chemical intermediates in the polymerization process.^1,2^ According to the latest information published by the International Agency for Research on Cancer (IARC), the carcinogenicity of these compounds is classified in terms of trichloroethylene in group 1A (carcinogenic to humans), tetrachloroethylene in group 2A (probably carcinogenic to humans), and carbon tetrachloride and chloroform in group 2B (possibly carcinogenic to humans). Therefore, due to the toxicity of halogenated hydrocarbons, trying to find an accurate, fast, and highly sensitive analysis method for biomonitoring these compounds in the human body is still important. One of the acceptable techniques for the bio-monitoring of halogenated hydrocarbon compounds is the use of specific biomarkers in urine.^3^ The non-metabolized form of chemical compounds usually has more specific biomarkers than their metabolite forms(s), because the metabolized compounds may be derived from other exogenous or endogenous compounds.^4,5^ The non-metabolized forms of chloroform, trichloroethylene, tetrachloroethylene and carbon tetrachloride in the urine sample as reliable and sensitive biomarkers have been measured using conventional techniques such as thin layer headspace, solid-phase extraction, and HS-SPME.^6–11^ Despite the eye-catching advantages of the SPME method, limited adsorption capacity and fiber fragility are some of the disadvantages.

The needle trap device (NTD) as one of the micro-extraction techniques can be employed in the active sampling, preparation, and desorption of workplace air pollutants in aqueous and gaseous matrices in one step and without consuming organic solvents.^12^ Moreover, the increased absorption capacity and resolving the needle fragility issue are advantages of the suitable design of adsorbent placement inside the needle.^13,14^ One of the key points of NTD is its flexibility in the employed adsorbent. Various adsorbents with interesting properties have been used in the NTD method.^12,15–17^ One of the best candidates for needle packing is the metal–organic framework (MOF) material. The footprint of MOFs can be seen in chemical applications such as separation, storage, catalysis, drug delivery, pollution analysis, and electrochemical applications such as supercapacitors, electrocatalysis, and sensors.^18–23^ The positive outlook comes from the diversity of structures, the flexibility of synthesis procedures, high surface area (up to 6000 m^2^ g^−1^), adjustable pores, selective adsorption, reusability, and suitable chemical and thermal stability as some of the advantages of MOFs.^24–27^ But MOFs suffer from a weakness compared to zeolites and activated carbons. The poor water stability and performance of MOFs in humid or aqueous environments is the most Achilles' heel of these materials which limits their employment in humid environments such as biological matrices. Various studies have been performed for resolving this issue. The employment of bi-metallic MOFs (bi-MMOF) is one of the ways to increase the stability. Bi-MMOFs have superior properties to mono-MMOFs due to the simultaneous placement of two different metallic cations as electron acceptors in the MOF structures. The stronger electrostatic attractions lead to powerful bonds and more active sites which cause the enhanced mechanical, hydrothermal and humid stability in wet environments. Ni^2+^ with a lower standard reduction potential makes it less likely for the metal center to react with water. Therefore, the water stability of Zn–MOF can be increased *via* doping with Ni^2+^.^28^

**Scheme 1:**
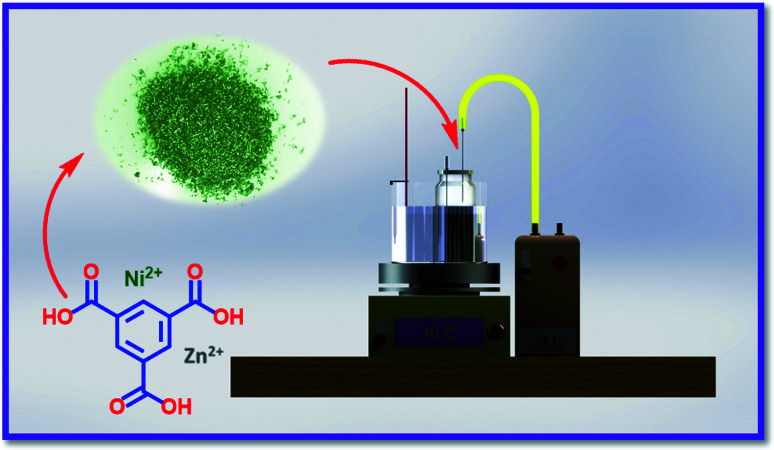
Designed setup for sampling of halogenated hydrogens in urine using the HS-NTD:Ni/Zn–BTC bi-MMOF.

To the best of our knowledge, there is no document on the use of Ni/Zn–BTC bi-MMOFs for bio-monitoring of halogenated hydrocarbons from urine samples by the NTD method. Herein, the Ni/Zn–BTC bi-MMOF as a water-stable absorbent was employed for sampling, extraction and determination of the non-metabolized halogenated hydrocarbon compounds (trichloroethylene, tetrachloroethylene, chloroform, and tetrachloroethylene) in the human urine using a dynamic headspace NTD-GC-FID (Scheme 1). To validate the proposed analytical procedure, the quantitation limit, detection limit, ranges, storage stability, carryover, and breakthrough volume were investigated. Eventually, the acceptable performance of the proposed Ni/Zn–BTC:NTD technique was examined in real urine samples.

## 2. Materials and methods

### 2.1. Chemicals and reagents

High purity trichloroethylene (99%), tetrachloroethylene (99.5%), chloroform (99.3%), and tetrachloroethylene (99.2%) as targeted analytes were purchased from Sigma Aldrich company. Moreover, Zn(ii) nitrate hexahydrate (Zn(NO_3_)_2_·6H_2_O; 99%), nickel(ii) nitrate hexahydrate (Ni(NO_3_)_2_·6H_2_O; 99%), and benzene-1,3,5-tricarboxylic acid (H_3_BTC; 95%) as reagent-grade materials for the preparation of the bi-MMOF absorbent were purchased from the Merck company (Darmstadt, Germany). The chemicals were used as received without any purification. Ethanol (C_2_H_5_OH; 99%) as the solvent was purchased from Merck company (Darmstadt, Germany). Pure nitrogen gas (99.9%) was obtained from Roham gas Co (Tehran, Iran).

### 2.2. Instrumentation

In this work, a flow sampling pump (model SKC 222-3; USA) with a flow rate of 1–200 mL min^−1^, a spinal needle (gauge 22: 0.71 × 90 mm; Kosan, Japan-Tokyo), and a Luer-locks syringe (Mina Tajhiz, Tehran) to inject the carrier nitrogen gas were utilized. A heater (TAT-94-1062, Tehran, Iran) was employed to adjust the temperature of the urine samples during the extraction process. Standard solutions were prepared in the 20 mL Teflon-silicone vials. Furthermore, a digital thermometer (Testoterm model, GmbH, Company) was utilized for online monitoring of the sampling chamber temperature. To determine and quantify halogenated hydrocarbons a gas chromatograph (Varian CP-3800) with a capillary column (CP7462: 30 m × 0.25 mm) coupled with a flame ionization detector (FID) was employed. The column temperature program started from 90 °C up to 180 °C with a rate of 10 °C min^−1^ and then was kept constant for 3 min. For thermal desorption of the halogenated hydrocarbons, the injector and detector temperature programs were adjusted in the range of 220–300 °C and 200 °C, respectively. For characterization of the prepared Ni/Zn–BTC MOF adsorbent the following apparatus was utilized: a Rigaku X-ray diffractometer (XRD) (Ultima IV; Japan) coupled with the transmission mode set at Cu K_α_ radiation in the 2*θ* range 5°–80° for the X-ray diffraction pattern. A microscope (MIRA3 TESCAN-XMU; Brno, Czech Republic) was used for recording FE-SEM images. A Fourier transform infrared (FT-IR) spectrometer (Perkin-Elmer GX FT-IR; USA) was used for recording FT-IR spectra patterns.

### 2.3. Preparation of the Ni/Zn–BTC bi-MMOF

In this procedure, the Ni/Zn–BTC bi-MMOF was prepared by a hydrothermal method with some modifications.^29,30^ For this goal, 0.46 g Ni(NO_3_)_2_·6H_2_O and 0.46 g Zn(NO_3_)_2_·6H_2_O as metallic cation sources were added in 15.0 mL of distilled water (solution A). Moreover, 0.42 g trimesic acid (H_3_BTC) ligand was dissolved into 15.0 mL of ethanol (solution B). Then, solution B was slowly added drop by drop to solution A under magnetic stirring. The combined solution was aged under magnetic stirring for 30 min. The prepared precursor solution was transferred into a Teflon-lined autoclave and heated at 120 °C for 24 h. Eventually, the obtained pale green crystals were accumulated from the solution and worked up with distilled water and ethanol (three times) for the removal of the unreacted raw materials from pores. Moreover, the Ni/Zn–BTC bi-MMOF crystals were aged at 80 °C for 12 h for the removal of the trapped solvent molecules in the pores and the activation process.

### 2.4. Preparation of an NTD

In this work, an NTD with a 22-gauge medical needle (OD and ID: 0.71, 0.39 mm, respectively) was used for packing and sampling of analytes. The needle was filled with 1.5 mg of the Ni–Zn:BTC bi-MMOF adsorbent and 1.0 mg crushed glass for avoiding pressure drop and blockage in the needle. It is noteworthy that the used crushed glasses didn't show any absorption for the halogenated hydrocarbon compounds in the pre-test experiments. The 15.0 mm of a needle was filled with the sorbent and crushed glass mixture and blocked using fiberglass with a length of 3 from two sides. The standby needle is inserted in the injection port of the GC for 2 h at 300 °C to remove the impurities and interference materials. Finally, the needle was placed in the injection port of the GC for the thermal desorption process through the passing of 3.0 mL nitrogen gas after extracting the halogenated hydrocarbons.

### 2.5. Pilot study

For the accomplishment of the pilot study, a stock standard solution (500 μg mL^−1^) of the targeted analytes (trichloroethylene, tetrachloroethylene, chloroform, and tetrachloroethylene) was prepared. In the extraction step using the NTD, 5.0 mL working standard solution (25.0 μg mL^−1^) and different amounts of NaCl were added to a 20 mL PTFE vial which was completely sealed by a Teflon-silicone and aluminum cap. The desired temperature of PTFE vials was provided by indirect heating (ban Mary) and monitored using a thermometer. An empty needle (size: 22.0) was placed in the liquid of the vial to prevent vacuum (see Scheme 1). Finally, the needle containing the extracted halogenated hydrocarbon compounds was placed in the injection port of the GC for the thermal desorption process.

### 2.6. Response surface methodology

The response surface methodology is a statistical method that can be used to evaluate the responses of several factors simultaneously with a few experiments. The purpose of its application is to find operating conditions or factor levels to optimally adjust the response variable. In fact, instead of using the trial and error method (spending a lot of time and money), the RSM methodology is used to get rid of these costs and achieve the optimal and reliable answer.^31^ In this study, the number of experiments was determined using the response surface methodology and Box–Behnken design (RSM-BBD).

### 2.7. Extraction condition

In this study, the extraction factors such as temperatures, times, and salt content were analyzed for determining the extraction efficiency of the halogenated hydrocarbons by the NTD filled with the Ni/Zn–BTC bi-MMOF adsorbent simultaneously. For this goal, the above-mentioned extraction factors were optimized using the RSM-BBD methodology at five temperature levels (25, 30, 40, 50, and 70 °C), five-time levels (10, 20, 35, 45, and 60 min), and three percent of salt additive levels (0.0, 7.5, and 15.0% w/v).

### 2.8. Desorption condition

In this research, to optimize the desorption parameters, the relationship between the injection port temperature of the GC device (200–300 °C) and desorption time (1–5 min) as independent variables, and the interaction of these two variables on the extraction efficiency of desired analytes were investigated. Then, the calibration curves were plotted for different desorption times. The optimal desorption time was determined at the minimum time with the largest peak area.

### 2.9. Breakthrough volume

The breakthrough volume (BTV) in the NTD method depends on the amount of packed adsorbent, the analyte affinity for the solid sorbent, the concentration of the analyte in the sample, and the passing volume of air through the needle trap. To investigate the breakthrough volume, two NTDs with similar properties of adsorbent length and pressure drop were connected in series. After extraction of the studied analytes, the NTDs were separated and the second NTD was placed in the injection port of the GC device for an examination of the desired analyte peaks.

### 2.10. Carryover

The carry-over effect was investigated using a needle trap packed with the Ni/Zn–BTC adsorbent with a high concentration of analytes (25.0 μg mL^−1^). After one step of the HS-NTD sampling and thermal desorption process, the needle was re-injected into the GC device according to the optimal desorption conditions to investigate the carryover on the Ni/Zn–BTC bi-MMOF absorbent.

### 2.11. Storage time

The storage duration of analytes in the adsorbent media after sampling is one of the most important issues in the development of the sampling method. In this study, the storage duration of the studied analytes in the Ni/Zn–BTC bi-MMOF adsorbent was investigated after 2, 4, 6, 8, and 10 days of sampling at a concentration of 25.0 μg mL^−1^. After sampling, both sides of the needles were closed with Parafilm and placed in a glass container and kept under two different conditions (ambient temperature and 4 °C).

### 2.12. Method validation

In this section, the repeatability, intermediate precision, ranges, detection limits, and quantitation limits were evaluated to validate the proposed method for headspace sampling and analysis of the halogenated hydrocarbons in the urine samples. The precision of the experiments was evaluated using repeatability and intermediate precision parameters, which were expressed by the relative standard deviation (RSD%). To determine the repeatability of the method, the RSD was calculated in three concentrations of analytes (10.0, 20.0, and 30.0 μg mL^−1^) with six replications per day (intraday) and five different days (inter-day, *n* = 30). The intermediate precision was determined by sampling three similar needles at one concentration (high level: 25.0 μg mL^−1^). The ranges of the studied analytes were calculated using the coefficient of determination of the calibration curve regression equations. To determine the value of detection limits and quantitation limits, the urinary concentrations of studied analytes were decreased to correspond to the signal-to-noise ratios of 1 : 3 and 1 : 10, respectively.

### 2.13. Extraction efficiency

To evaluate the extraction efficiency (EF%) for the proposed method, three different levels of studied analytes (10.0, 20.0, and 30.0 μg mL^−1^) were prepared in the urine matrix. Then, the extraction efficiencies of halogenated hydrocarbons at each level were calculated according to the following equation. Finally, the average extraction efficiency was calculated for each analyte.
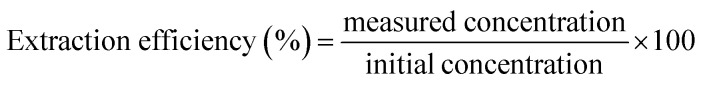


### 2.14. Real sample

Eventually, the feasibility of the HS-NTD:Ni–Zn–BTC:GC-FID method was analyzed for the extraction of the real urinary halogenated hydrocarbon samples under optimized conditions in the laboratory field. For this goal, urine samples were taken from six laundry workers post-shift with chronic exposure to halogenated hydrocarbons (Iran). Afterwards, the non-metabolized halogenated hydrocarbons in the real urine samples were extracted by the HS-NTD:Ni/Zn–BTC method under the optimal conditions (extraction and thermal desorption) and were analyzed three times with the GC-FID technique.

It is noteworthy that all experiments were performed in accordance with the “General Medical Council (GMC) Guideline”, and approved by the ethics committee at Bam University of Medical Sciences, with the code of ethics IR.MUBAM.REC.1399.065. Informed consents were obtained from human participants of this study.

## 3. Results and discussion

### 3.1. Characterization of the synthesized sorbent

**Fig. 1:**
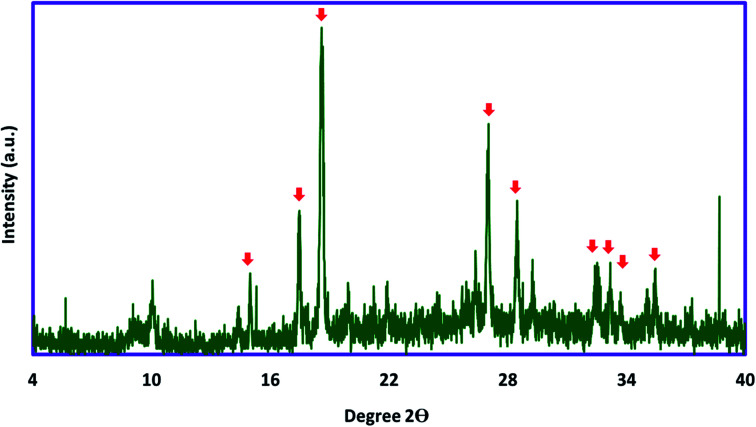
The PXRD pattern of the synthesized Ni/Zn–BTC bi-MMOF.

For investigation of the chemical and physical aspects of the absorbent, full characterization of the Ni/Zn–BTC bi-MMOF was carried out by FT-IR, PXRD, EDX, FE-SEM, and elemental mapping techniques. The powder X-ray diffraction pattern of the Ni/Zn–BTC bi-MMOF was recorded for examination of the purity and crystallinity of the absorbent (Fig. 1). The appeared characteristic peaks (especially 2*θ* = 17.6°, 18.7°, and 26.7°) in the PXRD pattern indicate isoreticular Ni– and Zn–BTC MOFs which has been reported in the previous documents. Moreover, this pattern proves the high purity and crystallinity of the synthesized absorbent.^29,32–34^

**Fig. 2:**
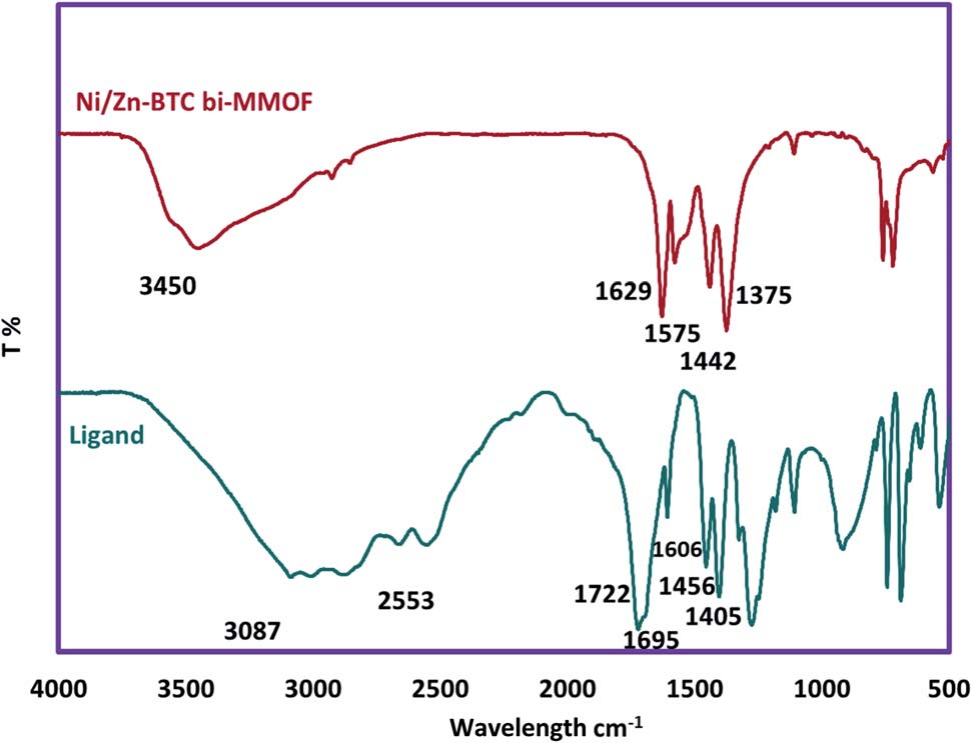
The FT-IR spectra of the ligand (H_3_BTC) and synthesized Ni/Zn–BTC bi-MMOF.

The FT-IR pattern of the Ni/Zn–BTC bi-MMOF was recorded for confirmation of bonding properties and functional groups. Fig. 2 exhibits the comparative FT-IR patterns of benzene-1,3,5-tricarboxylic acid (H_3_BTC) as the utilized ligand and prepared Ni/Zn–BTC bi-MMOF. The contribution of the carboxylic acid groups to the organization of the bi-MMOF structure can be justified by the absence of broad peaks (3087–2553 cm^−1^) in the MOF spectrum. The absence of a free carbonyl group peak at 1722 cm^−1^ in the MOF spectrum can be a strong argument for the participation of the ligand in the configuration of MOFs. Finally, the shifting of characteristic coupled bands from 1695–1606 and 1456–1405 to 1629–1575 and 1442–1375 cm^−1^, respectively, in the MOF spectrum can be related to the coordination of the free carbonyl groups with the metallic cations. These patterns are aligned with the reported documents.^29,30,35,36^

**Fig. 3:**
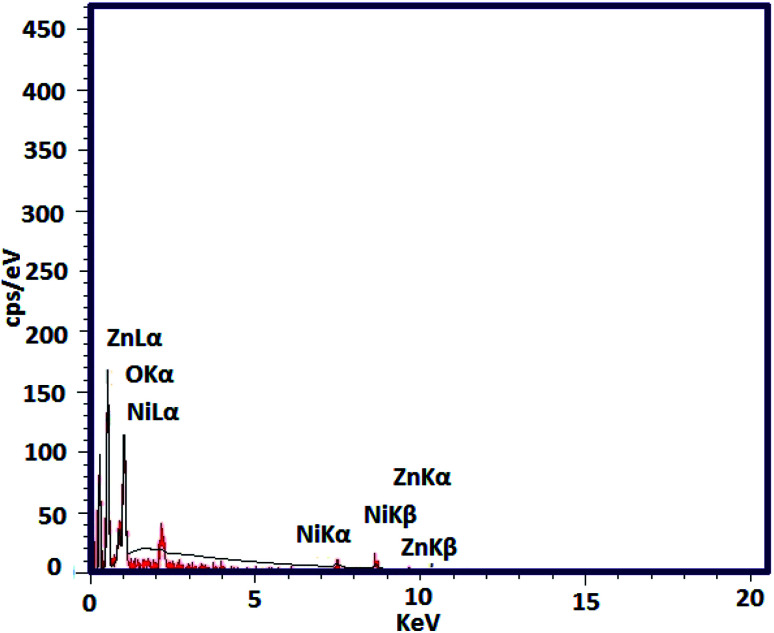
The EDX pattern of the synthesized Ni/Zn–BTC bi-MMOF.

**Fig. 4:**
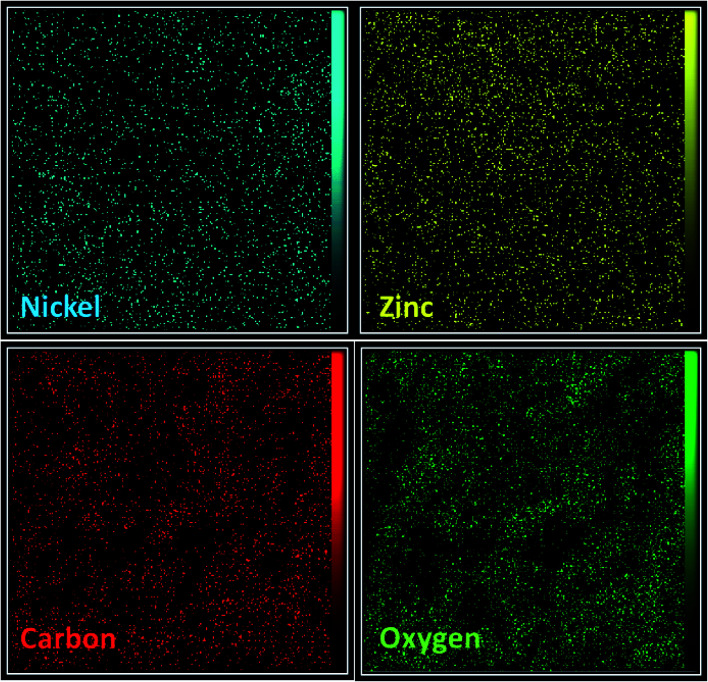
The EDX elemental mapping of the synthesized Ni/Zn–BTC bi-MMOF.

In the next step, EDX analysis was performed for examination of the involved elements in the final bi-MMOF configuration (Fig. 3). The obtained characteristic elemental peaks in the EDX pattern proved the simultaneous contribution of the Ni and Zn as involved metallic cations in the bi-MMOF structure. Furthermore, the recorded images of elemental mapping illustrated the uniform distribution of carbon, oxygen, zinc, and nickel elements in the bi-MMOF structure (Fig. 4).

**Fig. 5:**
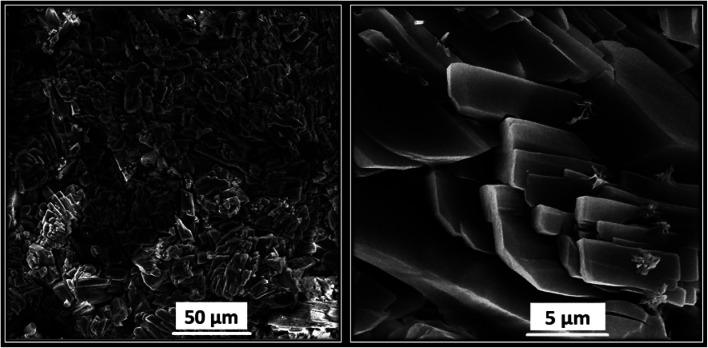
The FE-SEM images of the synthesized Ni/Zn–BTC bi-MMOF.

Lastly, the morphology of Ni/Zn–BTC bi-MMOF crystals was analyzed by FE-SEM. Fig. 5 illustrates the uniform and compacted cylindrical cubic microstructures of the synthesized Ni/Zn–BTC bi-MMOF. According to the images, the size of crystals can be estimated to be in the range of 5.0–10.0 micrometre nearly.

### 3.2. Extraction condition

#### 3.2.1 Extraction temperature

**Fig. 6:**
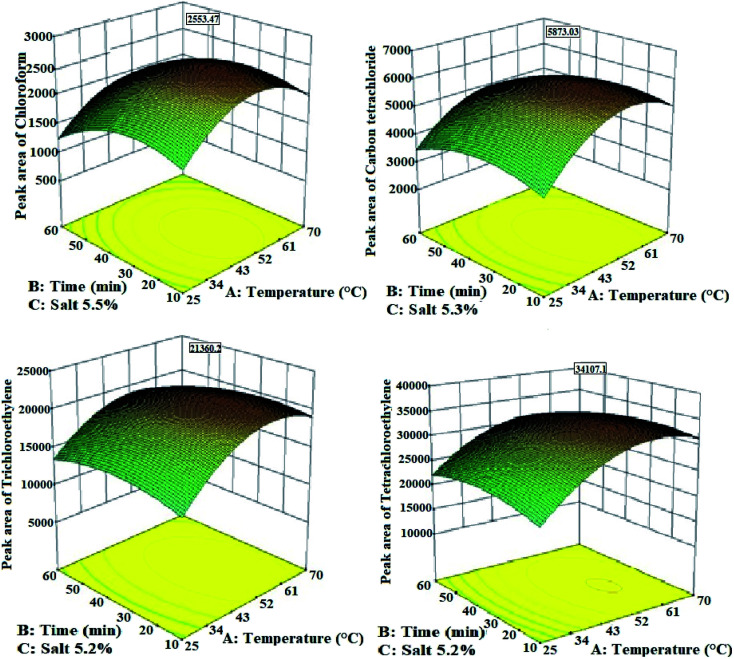
The effect of extraction parameters on the extraction efficiency of halogenated hydrocarbons from the urine samples by the HS-NTD:Ni/Zn–BTC bi-MMOF method.

Temperature parameter plays a key role in the headspace extraction method. It is noteworthy that a high extraction temperature can increase the diffusion of analytes in the gaseous phase and consequently reduce the adsorption rate by the solid adsorbent. To determine this effect on the extraction efficiency of urinary halogenated hydrocarbons, the effect of different temperatures (25, 40, 30, 50, and 70 °C) was investigated. According to Fig. 6, the highest extraction efficiency of targeted analytes is obtained in the temperature range of 51–56 °C. Moreover, the decreased extraction efficiency is obvious at higher temperatures (especially above 60 °C). Three reasons can be given for this result: first, increasing the extraction temperature leads to increased evaporation of analytes from the liquid matrix to the headspace of the sample and thus increases the extraction efficiency. Second, high temperatures can reduce the partition coefficients of the analytes between the gaseous phase (headspace) and the solid phase (adsorbent), because of the exothermic process of adsorption on the surface of the Ni/Zn–BTC bi-MMOF adsorbent. Third, the vapour pressure of water molecules in the headspace of the matrix can be increased with the temperature rising to higher points.^37^ In addition to the interference of the transfer of the analyte from the liquid sample to the headspace matrix, these molecules can compete with the other analytes for connection to the adsorbent surface. Therefore, they reduce the extraction efficiency.

The combination of the second and third effects may be much larger than the first effect. Therefore, the extraction efficiency of the analytes decreases with increasing the sampling temperature from a certain temperature.^37^ As a result, in this study, 56 °C was determined as the optimal extraction temperature of halogenated hydrocarbons from the urine samples by the HS-NTD method.

#### 3.2.2 Extraction time

The extraction time of urinary halogenated hydrocarbons was optimized. In this regard, the packed NTD with the Ni/Zn–BTC bi-MMOF adsorbent was inserted in the gaseous phase of the headspace for 10, 20, 35, 45, and 60 min, and quickly injected into the GC device for analysis. As can be seen in Fig. 6, the extraction efficiency of halogenated hydrocarbons reached its highest level in 30 min. Therefore, this time (30 min) was chosen and used as the optimal extraction time.

#### 3.2.3 The effect of adding salt

For the investigation of the salt content effect on the extraction efficiency of halogenated hydrocarbons from the urine samples, a range of 0–15.0% w/v of sodium chloride (NaCl) was added to the samples. The microextraction was performed by the HS-NTD method after a complete mixing process. The findings indicate that the addition of salt to the urine sample up to 5.5% w/v can increase the extraction efficiency of the desired analytes. But the extraction efficiency has remained unchanged at higher amounts of salt contents (Fig. 6). Therefore, this amount (5.5% w/v) was selected as the optimal amount of the salt additive to the urine sample for the extraction of halogenated hydrocarbons. The predicted values of optimal extraction temperature and time by the RSM-BBD methodology are shown in Table 1. These findings showed that the quadratic model can be acceptable for these data with a higher *R*^2^ value than the linear or 2FI models. The results of the ANOVA analysis proved that the extraction temperature can have the greatest effect on the extraction efficiency of halogenated hydrocarbons from the urine samples using the HS-NTD:Ni/Zn–BTC method (Table 1).

**Table 1:** Optimal conditions and results of the ANOVA test of extraction and desorption parameters of halogenated hydrocarbons by the HS-NTD:Ni/Zn–BTC bi-MMOF method

	Parameters/analytes	Chloroform	Carbon tetrachloride	Trichloroethylene	Tetrachloroethylene
Desorption variables	Optimal temperature (°C)	268.5	272.5	273.0	281.0
	Optimal time (min)	3.94	3.98	3.85	4.0
	*R*-Squared	0.89	0.88	0.89	0.92
	Adj *R*-squared	0.82	0.81	0.82	0.91
	SD	305.2	350.9	361.3	354.2
	CV	17.10	16.88	16.92	16.74
	PRESS	3.53 × 10^6^	4.67 × 10^6^	4.98 × 10^6^	4.79 × 10^6^
	*p*-Value	0.0025	0.0028	0.0017	0.0015
	Lack of fit	0.36	0.33	0.35	0.35
	Error bar	1.45	1.74	1.33	1.89
Extraction variables	Optimal temperature (°C)	51.65	53.17	54.68	56.57
	Optimal time (min)	27.04	27.74	28.87	29.58
	Optimal salt (w/v, %)	5.50	5.30	5.20	5.20
	*R*-Squared	0.95	0.94	0.92	0.93
	Adj *R*-squared	0.89	0.86	0.84	0.85
	SD	204.95	496.65	1794.14	2847.85
	CV	12.15	12.12	11.78	11.57
	PRESS	1.251 × 10^6^	9.357 × 10^6^	1.197 × 10^8^	3.017 × 10^8^
	*p*-Value	0.0006	0.0015	0.0016	0.0017
	Lack of fit	0.82	0.71	0.72	0.72
	Error bar	0.33	0.41	0.42	0.39

There was no significant difference between modeling data and experimental results obtained under the optimal conditions. For example, the desorption temperature of chloroform is 268.0 ± 1.3 °C in experimental data, and 268.0 ± 1.5 °C in modeling data (*n* = 3, *p* > 0.05).

From a comparative perspective, the optimal salt content, extraction temperature, and extraction time of the urinary halogenated hydrocarbons by the HS-SPME method which has been reported by Poli *et al.* are 36.0% w/v, 22 °C and 30 min, respectively.^10^ Moreover, the above-mentioned parameters for the extraction of the urinary halogenated hydrocarbons using a SPME fibre coated with the Al_2_O_3_:TiO_2_ adsorbent which has been reported by Farhadi *et al.* are 4.0% w/v, 50 °C and 15 min, respectively.^9^ In another study that has used the SPME fiber coated with the carbon nanotube sorbent for the sampling of these analytes, the optimal amount of salt, extraction temperature and time were 0.1 g mL^−1^, 60 °C, and 35 min, respectively.^11^

### 3.3. Desorption conditions

As it is known, the separation of the analytes from the adsorbent in the extraction methods is performed in the injection port of the GC device. Therefore, optimization of the device conditions is very important. The thermal desorption time should be selected based on the targeted analyte vapour pressure, the thermal stability of the adsorbent, and the adsorbent affinity for the analyte. Thus, at low temperatures, thermal desorption cannot be completely performed and causes less estimation of the actual concentration of analytes and moreover causes the carry-over effect on the adsorbent. On the other hand, the choice of high temperatures can degrade the packed absorbent structure and appearance of interfering peaks related to the adsorbent components. Therefore, in this work, the effects of required temperature and time were optimized for thermal desorption of analytes. For this purpose, the separate effect of the two parameters on the sampler performance and their interaction were investigated using the RSM-BBD methodology. The predicted values of optimal desorption temperature and time and other related parameters to the model by RSM-BBD are presented in Table 1. The quadratic model was considered as the best model due to the lower standard deviation value, acceptable *R*-squared values, and low PRESS value for all studied analytes. The value of *R*^2^ calculated in this model is between 0.89 and 0.92, which indicates the appropriate response of the model.

**Fig. 7:**
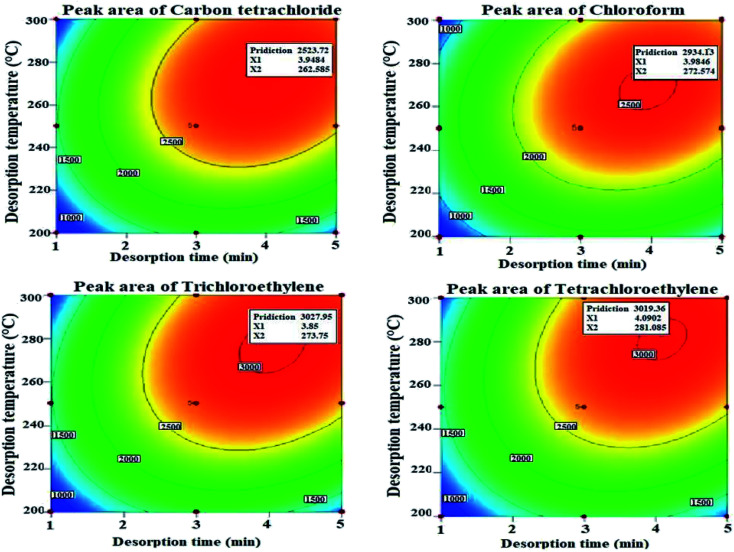
Effect of time and temperature on the thermal desorption of urinary halogenated hydrocarbons with the Ni/Zn–BTC:HS-NTD:GC-FID method.

The obtained results showed that the coefficient of determination of temperature and desorption time is significant in the proposed model (*R*^2^ = 0.89–0.92). The highest peaks appeared in the range of 268–281 °C and 3–4 min due to the interaction of the two parameters of temperature and desorption time, respectively, which is consistent with other similar studies.^9–11,38^ According to the results, increasing the temperature and desorption time to a certain range has increased the performance of the sampler, while a further increase in temperature and desorption time has reduced the thermal desorption performance of the studied analytes (see Fig. 7).

### 3.4. Breakthrough volume

In this regard, the sampling was performed at a flow rate of 3.0 mL min^−1^ and 25.0 μg mL^−1^ analytes for 8 h. Based on the findings, the peak area of the analytes is increased with increasing sampling time. But, the BTV was not observed even after 8 h of sampling with the proposed HS-NTD:Ni/Zn–BTC method.

The results showed that the Ni/Zn–BTC adsorbent had a high adsorption capacity to adsorb halogenated hydrocarbons.

### 3.5. Carryover

The results showed that the carry-over effect for each analyte is different and also depends on the amount of analytes absorbed on the adsorbent. According to the result, the remained percentage of analytes in the proposed adsorbent for the chloroform, trichloroethylene, tetrachloroethylene, and carbon tetrachloride was 0.01, 0.03, 0.03, and 0.02%, respectively. From a comparative point, Poli *et al.* have reported a headspace sampling of halogenated hydrocarbons in urine at 280 °C for 5 min by SPME with the carboxy-polydimethylsiloxane (PDMS) fiber without observing the carry-over effect which is comparable to the results of the present study.^10^

### 3.6. Storage time

The results of the levels of recovered analytes after 2, 4, 6, 8, and 10 days were compared with the result of sample analysis immediately after sampling and the amount of analyte reduction was determined so that the average peak area of each analytes was compared with their peak area in the control needle (analyzed immediately after sampling). Analysis of stored needles at 4 °C showed that there was no significant difference between the concentrations of halogenated hydrocarbons in control needles and those stored after 10 days (*p*-value > 0.05). As a result, halogenated hydrocarbons sampled using the NTD packed with the Ni/Zn–MOF adsorbent can be stored in the refrigerator for at least 10 days. Moreover, the peak area of the studied analytes in the stored needles at 25 °C after 2 days did not differ significantly compared to the control values (*p*-value > 0.05). But the concentration of studied analytes in the stored needles at 25 °C after 5 days was decreased by 40%.

### 3.7. Method validation

In this study, a calibration curve was plotted for the range of 1 to 16 000 ng L^−1^. The linearity of the calibration curve was determined according to the coefficient of determination (*R*^2^: 98–99).

Furthermore, according to the findings, the detection limit values of the urinary halogenated hydrocarbons sampled by the proposed HS-NTD:Ni/Zn–BTC:GC-FID method for chloroform, trichloroethylene, tetrachloroethylene, and carbon tetrachloride were estimated to be 1.1, 1.02, 1.1, and 1.04 ng L^−1^, respectively. The quantitation limit values for the mentioned analytes were determined to be 2.3, 2.01, 2.4, and 2.05 ng L^−1^, respectively. Furthermore, the linearity values were estimated to be 160 000 ng L^−1^ for the urinary halogenated hydrocarbons. From the comparative view, the detection limits of halogenated hydrocarbons in the urine matrix using the headspace SPME method have been reported to be in the range of 60.0 to 500.0 ng L^−1^.^9^ In another study, Poli *et al.* have reported the detection limits of urinary halogenated hydrocarbons using the headspace SPME:GC-MS method in the range of 5.0 ng L^−1^.^10^ Furthermore, the reported detection limits and quantitation limits to determine the concentration of BTEXs in the urine samples by the HS-SPME:MIL-100–Fe:GC-FID method were in the range of 0.0001–0.0005 and 0.0003 to 0.0014 μg mL^−1^, respectively.^39^ Moreover, in the study of Tajik *et al.*, the detection limit and quantitation limit levels of urinary volatile organic compounds sampled by the HS-Cool Fiber-SPME technique were determined to be in the range of 0.02–0.07 and 0.07–0.2 ng mL^−1^, respectively.^40^ A comparison of the results of this study with the other similar studies proves that the proposed method has lower detection limits and quantitation limits and wider ranges than other previous studies. Therefore, the HS-NTD:Ni/Zn–BTC:GC-FID can be used as a highly sensitive method to determine the halogenated hydrocarbons in the urine samples.

One of the important parameters that should be considered in the development of extraction methods is the repeatability and intermediate precision of the proposed method. Repeatability and intermediate precision indicate the precision of the used method, which is usually expressed in terms of the relative standard deviation (RSD%). Table 2 exhibits the repeatability and intermediate precision results of the proposed method. According to the results, the repeatability (<4.8%) and intermediate precision (<8.3%) are in the acceptable range for the NTD packed with the Ni/Zn–BTC bi-MMOF adsorbent. In this regard, the reported RSD for the repeatability of halogenated hydrocarbon sampling by a needle trap packed with the UIO-66:NH_2_ adsorbent in the air matrix was in the range of 2.3–10.2%.^12^ In another study, the reported RSD for intermediate precision using an NTD packed with the UIO-66 adsorbent for sampling aromatic hydrocarbons from the air was estimated to be less than 14.3%.^41^ Moreover, this parameter was reported to be less than 6.5% for the sampling of the halogenated hydrocarbons in urine samples by the HS-SPME method coupled with a fiber coated with the Al_2_O_3_–TiO_2_ composite.^9^ Therefore, it can be concluded that the proposed needle trap sampler offers satisfactory repeatability and intermediate precision results for commercialization.

**Table 2:** Results of repeatability and intermediate precision of sampling and analysis of urinary halogenated hydrocarbons by the HS-NTD:Ni/Zn–BTC:GC-FID method

Analytes	RSD% for a NTD at different concentrations (μg mL^−1^)			Error bar	RSD% for different NTDs at a constant concentration (25.0 μg mL^−1^)			Error bar
	10	20	30		NTD1	NTD2	NTD3	
Chloroform	2.3	2.3	2.9	0.1	5.6	4.9	6.1	0.2
Trichloroethylene	1.2	2.7	4.3	0.2	7.3	6.9	8.1	0.3
Tetrachloroethylene	2.7	1.4	1.4	0.1	8.3	7.1	5.5	0.2
Carbon tetrachloride	4.8	2.6	3.3	0.2	7.8	5.1	6.8	0.2

**Fig. 8:**
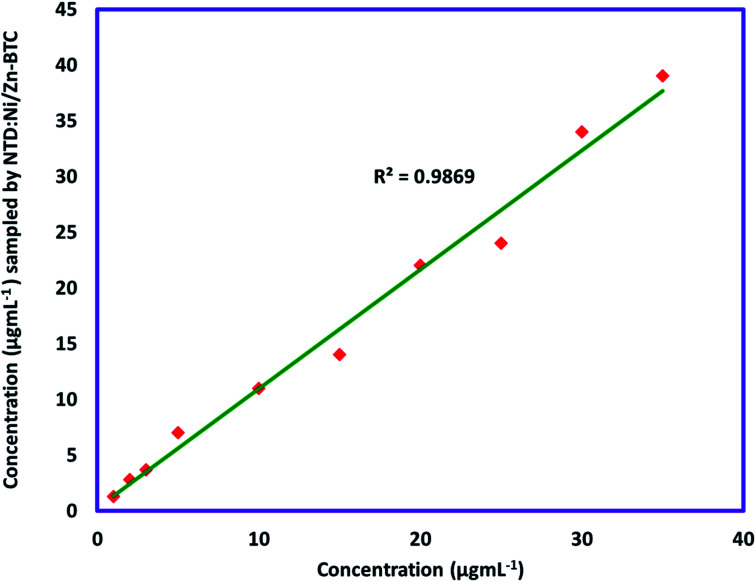
Comparison between the results of sampling of chloroform by NTD:Ni/Zn–BTC with spike samples.

The accuracy of the method was evaluated by comparing the results of NTD:Ni/Zn–BTC with spike samples of known concentration. Comparison of the results (Fig. 8) showed that there is a good correlation between the two methods (*R*^2^: 97–99%).

### 3.8. Extraction efficiency

The obtained results showed that the microextraction efficiencies of urinary halogenated hydrocarbons using the Ni/Zn–BTC bi-MMOF adsorbent for chloroform, trichloroethylene, tetrachloroethylene, and carbon tetrachloride were 91.40, 91.57, 90.87, and 91.00%, respectively. These results are better than or similar to the previously reported results.^9–11,39,40^

### 3.9. Real sample

In order to evaluate the application of this method in the field, the efficiency of the proposed method for measuring trichloroethylene and perchloroethylene in the upper urinary space of 6 laundry workers after the end of the shift was evaluated.

Based on the results presented in Table 3, the concentration of halogenated hydrocarbons in real urine samples extracted using the Ni/Zn–BTC bi-MMOF method was estimated to be in the range of 4.3–6.2 μg L^−1^.

**Table 3:** The concentration of halogenated hydrocarbons in the real urine samples of laundry workers

Analyte	Mean concentration (μg L^−1^)	RSD (%)	Error bar
Trichloroethylene	4.8	6.2	0.2
Tetrachloroethylene	4.3	5.9	0.3

## 4. Conclusion

In this study, the Ni/Zn–BTC bi-metallic MOF was introduced as a water-stable absorbent for the monitoring of non-metabolized halogenated hydrocarbon compounds in the urine matrix, for the first time. The proposed adsorbent was employed in a dynamic headspace-needle trap device as an impressive and moisture stable absorbent in the laboratory and real-time conditions. The response surface methodology and Box–Behnken Design software were utilized for optimization of the extraction and thermal desorption parameters. The proposed technique offers a satisfactory extraction efficiency to determine the trace levels of halogenated hydrocarbon compounds in the urine matrix. The obtained results proved that this procedure can be an eco-friendly method with suitable repeatability and intermediate precision and acceptable specificity under the free-organic solvent conditions. According to the results, the optimal extraction time, temperature and salt content were 30 min, 57 °C and 5.5%, respectively. Moreover, the thermal desorption parameters (time and temperature) were determined to be 4 min and 285 °C, respectively. The detection limit values for chloroform, trichloroethylene, tetrachloroethylene, and carbon tetrachloride were examined to be 1.1, 1.02, 1.1, and 1.04 ng L^−1^, respectively, which illustrates the suitable specificity of the method. The results of this study showed that the detection limit of the proposed method is lower than the allowable biological exposure indices (BEIs) of the studied compounds; eventually, the introduced adsorbent accomplished bio-monitoring of non-metabolized halogenated hydrocarbon compounds in the real urine samples. The findings illustrated that the HS-NTD:Ni/Zn–BTC:GC-FID technique can be employed as a reliable procedure for monitoring of non-metabolized halogenated hydrocarbon compounds in urine samples.

## Conflicts of interest

The authors declare no conflict of interest regarding the publication of this article.

## Supplementary Material
